# Foreign Hospital Practice

**Published:** 1828-04-01

**Authors:** 


					1828] ( 457 )
^ci-t'jScojje;
CIRCUMSPECTIVE REVIEW.
" Ore trahit quodcunque potest, atque addit acervo."
[26th JANUARY, 1828.]
FOREIGN HOSPITAL PRACTICE.
1. PENNSYLVANIA HOSPITAL.
On Delirium Tremens.
[By Dr. Coates."]
This mysterious and dangerous disease is
very common in the United States of
America, where the excessive- use of
ardent spirits is but too prevalent. The
transatlantic physicians, therefore, have
ample opportunities of studying the phe-
nomena, the pathology, and the treat-
ment of mania a potu, as it has been
called. Dr. Coates of Philadelphia, has
lately published a very extended and in-
teresting memoir on this subject, in the
North American Medical and Surgical
Journal, from which we shall make some
extracts in the present paper. We shall
begin with the conclusions to which our
author has come, from extended obser-
vation, reading, and reflection. These
ai'e in the form of aphorisms :?
" 1. The disease is a delirium, and not
a mania ; and this distinction should be
attended to, both for medical and legal
Reasons.
" 2. It consists in aheightened activity
?f the sensorium ; and this appears to
arise from the generation, in that organ,
?f an unusual vital power, which is not,
as in common, exhausted by the narcotic
poisons habitually used. This is not con-
sidered as an hypothesis, but the expres-
sion of a fact existing in nature.
" 3. The delirium may be combined
}vith other diseases and injuries, situated
in many different parts of the body.
" 4. Whenviolent, it obscures and ren-
ders imperceptible most of the symptoms
of the co-existing disease.
" 5. It is doubtless necessarily accom-
panied, as all vital excitements are, with
an unusual amount of the circulation of
the blood in the organ affected ; and is,
from this cause, sensibly influenced by
cups, blisters and emetics. It is not so
far checked by the use of emetics as to
render these advisable as a leading means
of cure. It is not sufficiently under the
control of the general circulation to be
cured by venesection, or to be sensibly
relieved by it without such an exhaustion
as is highly dangerous to life.
" 6. It is entirely and absolutely under
the control of opirnii; although the fevers
and other diseases which are liable to
accompany it may be by no means so.
" 7. It admits of very large doses of
opium, which are not productive, either
at the time or subsequently, of any in-
jurious consequences, provided they are
not repeated after a tendency to sleep is
evinced.
" 8. The patient must sleep or die.
There is no alternative. Yet the phy-
sician should personally watch the effect
of very large doses of opium.
" 10. Purgatives are of 110 use in this
delirium ; but it is necessary to prevent
costiveness subsequently to the adminis-
tration of opium. Purgatives may be
necessary for diseases which exist at the
same time; but when this is the case,
they are, in general, most advantageously
postponed till after sleep has been ob-
tained.
"II. Gentle stimulants are frequently
useful during the convalescence; but these
should not resemble ardent spirits ; and
an excellent and sufficient one is capsi-
cum. Nor should any ardent spirits,
unless indicated by peculiar circum-
stances, be given during the paroxysm."
From the second aphorism, it will
be seen that Dr. Coates considers " a
heightened activity of the sensorium," as
the pathological condition of the brain,
Vol. VIII. No 16. 3F
458 Periscope ; on, Circumspective Review. [Jan. 26
in delirium tremens, though the disease
may be complicated with, or produce an
increased activity of the circulation, or
inflammatory action in the organ. We
believe this pathology to be as near the
truth as any that has yet been adduced,
and therefore we shall not dwell on this
part of the investigation, but proceed, at
once, to the treatment.
In the United States, this is a combi-
nation of several modes of practice, vary-
ing with the existing condition of the
patient. This is probably the best?but
still, as Dr. Coates observes, it would be
desirable to have some fixed principle for
our guidance in pure and unequivocal
cases. Thus it would be proper to have
made up our opinion whether the disease
be inflammatory or nervous, for if we
proceed on the former principle, we shall
do much mischief. If the disease be com-
plicated with other affections, as bilious
fever, pulmonic inflammation, &c. we
should modify our practice accordingly.
Bred up in the Pennsylvania Hospital,
Dr. Coates' first experience was with the
stimulo-narcotic treatment?the narcotic,
however, being in much smaller doses
than are now employed. Our author
was shocked with the mortality that ob-
tained. He saw, for example, three men
die in succession of delirium tremens,
who came in with fractures.* Those
who recovered, were invariably observed
to fall into a long and quiet sleep, as a
prelude to the cessation of the disease.
Those who died never experienced this
" solace of their woes," before disso-
lution.
" A patient belonging to the
theatre, was referred to my charge, during
a delirium tremens of extreme violence.
From a form at the commencement en-
tirely intermittent, his delirium had be-
come continued, increased greatly in vio-
lence, and resisted the action of very
large doses of opium, carried, in one
evening, beyond the amount of twenty
grains, and accompanied by considerable
quantities of alcoholic drinks. In this
? situation, his pulse x-apidly declining,
while the delirium was regularly increas-
ing, and after the disease had continued
upwards of a week, I judged that, ac-
cording to ordinary experience, the pa-
tient must certainly die, unless he ob-
tained sleep. I gave him six grains of
powdered opium every hour, visiting him,
and carefully watching the effect of the
sedative at every dose. It was not till
thirty-nine grains had been thus adminis-
tered, that a decided tendency to sleep
was observed. Rest, however, not being
obtained, another dose was given, making
forty-five grains ; and full sleep was pro-
duced within another hour. Next morn-
ing, the patient awoke perfectly rational.
Delirium returned, in a slighter degree,
for several nights following ; and minor
doses of opium continued to be given
for a long time after ; longer than I now
ever find necessary. But the patient's
life was saved; and he recovered suffi-
cient strength to struggle with a long
and severe surgical disease, to regain his
health, and return to his professional
employments. He subsequently died of
yellow fever, at New Orleans."
Another patient recovered by the ad-
ministration of 400 drops of Laudanum
in five hours. The impression made by
these two cases induced Dr. Coates to
act afterwards on the principle " that
SLEEP MUST BE OBTAINED AT ALL HA-
ZARDS and this, we are informed, led to
a success which, as time advances, has
more strongly confirmed him in the belief
of the propriety of the said principle.
Wherever time has been allowed for the
administration, by repeated doses, of a
large amount of opium, this practice was
always successful in curing the delirium;
In some cases, the patients ultimately
died of inflammation in some viscus, but
this does not impeach the principle laid
down. The following extract will give a
sufficient idea of Dr. Coates' practice.
" To produce any impression of what-
ever kind, during this disease, opium
must be given in doses increased enor-
mously beyond those which are requisite
for ordinary purposes. Five or six grains
alone, are, in a case of any severity, ab-
solutely a nullity?they will not drive
away a single spectre. The successful
amount with me, has generally bqen
from twelve, to twenty-eight, or thirty
grains ; but in an honest judgment, I can
assign to it 110 limit. I have never seen,
read of, or heard of, an instance in which
it was productive of any harm."
* Was not this disease the " trau-
matic delirium" of Dupuytren, des-
cribed in our last Fasciculus, page, 435.
J828] Hydatids of the Liver cured by Puncture. 459
" The only rule by which I would be,
and have always been guided, is the fol-
lowing. A certain effect is to be pro-
duced, coute qui coute; and we must go
on exhibiting opium in considerable doses,
at such short intervals as are sufficient to
Permit its accumulation in the primae vise,
until enough has been taken to produce
sleep. I have generally given it every
hour; but when sleep appears actually
approaching, a somewhat longer interval
may be allowed, to ascertain the fact,
without much hazard of defeating the
plan of cure. If the case be a slight
one, I have left doses amounting to five
or six grains with the attendants, di-
recting them to proceed with it, either
until sleep was produced, or the medicine
had all been taken. Upon the failure of
one trial like this, a much larger amount
must be employed; and where consi-
derable doses are given at every time, it
indispensibly necessary that the phy-
sician should superintend their effects
biinself, visiting the patient after every
fresh administration, and watching for
any disposition to drowsiness. Where
this has not yet appeared, there is not
the least danger to be apprehended ; as
we believe it may be safely denied that
there is a case on record in which any
injury was sustained."
Dr. Coates makes many judicious re-
marks on various other modes of, treat-
ment that have been recommended, and
also on some auxiliary remedies that may
he occasionally employed ; but these we
shall pass over. He, also, gives the cor-
roborative testimony of several respect-
able American physicians, as to the
efficacy of large doses of opium in deli-
rium tremens?a practice which we be-
lieve to be the best and the safest that can
he followed in this dangerpus malady.
2. HOTEL DIEU.
Cas e of Hydatids of the Liver cured
by Operation. By M Martinet.
^t is now well known that hydatids, whe-
ther they be morbid growths or living
animals, invade almost every structure in
the human body. They vary according
t? the part in which they grow. Those
?f the liver are inclosed in a cellular or
fibrous cyst, developed at the expense of
the cellular tissue of the organ, and are
found floating in a liquid, sometimes se-
rous and clear?sometimes puriform and
turbid ; the latter probably from inflam-
mation occurring in the parietes of the
cysts. In proportion as the cysts expand,
the larger hydatids burst?their debris
become mixed with the fluid?and the
fluid itself acquires a turbid appearance.
We are nearly in the dark as to the causes
of these, hydatid formations in the liver.
They have been observed more frequently
after contusions of the right hypochon-
drium than under other circumstances ;
but these cases are far from proving the
etiology of the disease. Like all other
prganic diseases of this viscus, hydatids
produce little or no inconvenience till
their size begins to disturb the function
of the organ, and occasion an enlarge-
ment of the right hypochondriac region.
Even then, it is next to impossible to di-
agnosticate with.any degree of certainty.
When there is a prominence, accompanied
by a fluctuation, we may conjecture, with
some degree of probability, that there is
either an hydatid formation, or a collec-
tion of purulent matter?in either of
which cases we imagine there can be
little objection to an operation. We shall
now proceed to the case under conside-
ration.
Case. A ship-painter, named Damenge,
aged 20 years, of strong constitution, and
who had enjoyed habitual good health,
with the exception of some attacks of
colic, fell from a stage, on the 26th April,
1827, to a distance of ten or twelve feet,
and was picked up insensible. On the
next day, a slight yellow tint was per-
ceived on his countenance, which soon
spread over the whole surface of his body;
but, as his health was not affected, he
returned to work on the 28th of the same
month. On the 30th, he felt pain in the
right hypochondrium, and he could not
lie on either side in bed. The pain in
his side was accompanied by a retraction
of the corresponding testicle, to which
were added thirst and fever. This state
continued till the 3d of May, when he
was received in the Hotel Dieu.
There was now very little fever?a
slight yellow tint pervaded the surface
of the body?tongue scarcely white?no
head-ache?no pain in the right shoulder
3 F 2
A
A
460 Periscope; or, Circumspective Review. [Jan. 2(3
?constipation during the last four days.
In the right hypochondrium, there was a
tumour, of irregular form, but not tabu-
lated, extending from the ensiform car-
tilage to the edges of the false ribs, (which
were a little raised) downwards to below
the umbilicus. Pressure produced but
little uneasiness, and the sense of touch
discovered the presence of several bodies,
somewhat hard, salient unequal, andpul-
sative. The pulsation was considered as
communicated from subjacent vessels. In
several points, an obscure fluctuation was
perceptible. Percussion elicited a very
dull sound from the whole of the upper
and right side of the abdomen, down as
low as the right flank. On striking va-
rious points of the abdomen with one
hand, while the other was kept steadily
on the tumour, no shock was communi-
cated to the latter. The application of
the ear to the parts communicated no
sound?but rather an absence of all sound.
Venesection?loiv diet.
On the 4th May, a very fine trocar was
introduced into the part where fluctuation
had been least equivocal, and a cupping-
glass applied. A few drops of limpid
fluid escaped by the aperture. During
the succeeding days, the health of the
patient improved, and the icterus dimi-
nished. A caustic of potassa fusa was
applied on the most salient point, under
the false ribs?this was repeated on sub-
sequent days. Mean time, the tumour
seemed to get flatter, and lessened in vo-
lume. On the 15th May, the eschars fell
off, and, on the 19th, the tumour opened
spontaneously into the bottom of the
wound, and discharged a great quantity
of yellow fluid, containing numerous hy-
datids. The diameter of the opening
was about half an inch. Three basins
were quickly filled with this discharge,
and innumerable hydatids, of various sizes,
up to that of an egg, (elongated in pas-
sing) were disgorged, with great dimi-
nution of the volume of the abdomen.
The majority of these acephalocysts pre-
sented transparent coats, two or three in
number, containing an aqueous fluid,
which soon turned opake, on being ex-
posed to the air. During the next few
days, there was a considerable discharge
of hydatids, and the patient continued
free from fever. Some mild fluids were
injected, from time to time, and returned
nearly colourless. The hydatids that is-
sued on the 23d of May were of a yellow
colour, apparently tinged with bile. The
tumour had greatly subsided, and a pretty
tight bandage was kept round the abdo-
men. The injection returned this day,
rather fetid in odour. 26th. The injec-
tion returned much less fetid. In the
course of the following days, an injection,
composed of decoction of bark and solu-
tion of chloruret of lime, was employed,
and produced some smarting pain. On
the 5th June, a slight diarrhoea came on,
which was remedied by a more restricted
diet. On the 11th June there could not
be more than four ounces of injection
thrown into the cyst; and, by the 22d of
the same month, it was impossible to in-
troduce any thing. 5th July. A slight
discharge of purulent matter takes place
every day, and the wound is closing fast.
On the Gth July, the discharge presented
the odour of feculent matter ; and, next
evening, some fragments of pease, eaten
at dinner were unequivocal in the dis-
charge from the wound. From the Sth
till the 20th of the same month, portions
of undigested aliment presented them-
selves among the discharge, which was
always of a stereoraceous odour. Sutures
were tried to close the fistula, but they
failed. On the 30th, the patient left the
hospital, in perfect health, with the in-
convenience, only, of a small stercora-
ceous fistula. In the early part of August,
he presented himself at the Hotel Dieu,
with the fistula completely cicatrized, and
his health excellent.
We consider the above as a very inte-
resting case, the details of which are
stated with equal modesty and perspicuity.
It may tend to diminish the fear of giving
vent to hydatid tumours, (especially in
the cautious way practised on this occa-
sion) which has been inculcated by various
surgical writers, down even to the present
day.
Stricture of the Lachrymal Duct.*
During Mr. Teale's attendance on M.
Dupuytren's practice at the Hotel Dieu,
* Mr. Teale. Ed. Journ. Jan. See
also an excellent paper on the anatomy
of these parts, in the November Number
of the Medical Repository, by Mr. Reeve.
1828] Comparative Success in Amputations. 461
he was much gratified at the success of
that surgeon's treatment of stricture of
the lachrymal duct. The operation con-
sists in the simple insertion of a metallic
tube into the lachrymal canal, over which
the integuments are to be healed. M.
Dupuytren is said to be successful in
nineteen out of twenty cases. The ca-
nula, which is of silver, gold, or platina,
is one inch in length?its upper opening
measures one tenth of an inch?the tube
gradually tapering from its upper to its
lower opening, the diameter of which
measures one twentieth part of an inch.
The lower opening is placed obliquely,
and the upper one is surrounded by a
small rim, to prevent its sinking too low
into the duct. The surgeon having punc-
tured the lachrymal sac with the point
of a bistoury, inserted close below the
tendon of the orbicularis palpebrarum,
leaves the instrument there as a director,
till the canula is insinuated along it into
the lachrymal canal. The bistoury is
then withdrawn?slight pressure is made
upon the upper part of the canula, so as
to imbed it in the canal?and the ope-
ration is completed. The patient is then
directed to blow down the nose, at the
time that pressure is made upon the nos-
trils, by which the tube is freed from any
blood that may have accumulated in it,
while the air issues out at the incision,
shewing the free communication esta-
blished between the nostril and sac. Mr.
Teale has detailed some eases of his own,
successfully treated upon M. Dupuytren's
plan.
On the comparative Results of Am-
putation in Military and Civil
Hospitals, and on the Field of
Battle. By M. Sanson, Surgeon of
the Hotel Dieu, Paris.
It is a common remark, that amputations
succeed better in military hospitals, and
on the field of battle, than in civil hos-
pitals. This is true, generally speaking.
But what, says M. Sanson, are we to
conclude from it ? Is this superior suc-
cess owing to greater dexterity in the
operator, or a better mode of operating,
or more carc afterwards ? We agree with
him, that such a conclusion would be un-
fair, as well as erroneous. The difierence
of results, then, he thinks, are to be
sought in other circumstanccs, as the
age, sex, state of health, and moral dis-
positions of the patients.
1. Age. Soldiers, generally speaking,
are young, or, at all events, in the prime
of life. Those operated on in civil hos-
pitals are, for the most part, beyond the
medium age of soldiers?and exhausted by
poverty, intemperance, or over-exertion.
2. Sex. A good number of amputated
patients, in civil hospitals, are females?
and, according to our author, females who
have deranged, or completely obstructed
menstruation, which operates injuriously
on their health, and renders the success
of amputation more problematical. This,
we think, is rather an unsatisfactory
3. State of general Health. Unques-
tionably, the soldier has an immense ad-
vantage, in this respect, over the wretched
objects which undergo amputation in civil
hospitals.
4. Moral Dispositions. The soldier,
says, M. Sanson, marches into the field
of battle at the risk of life, and, therefore,
thinks himself well off with the mere loss
of a limb. In other respects, he is free
from care or anxiety. A pension, or a
domicile in some place under government,
insures him an honorable existence. He
submits not only cheerfully to amputation,
where it is necessary, but even cuts jokes
on the occasion. If he loses a leg, he
saves half the expense of shoes and stoc-
kings ! The moral frame of the artisan's
mind is in a very different condition, when
doomed, in a civil hospital, to the loss
of a limb. Worn out with penury and
disease before he enters the hospital, he
sees all his hopes of a cure vanish into
air, and finds himself obliged to undergo
an operation, that deprives him of a mem-
ber which he can ill spare, in gaining a
subsistence, or maintaining a family.
It is no wonder that this depressed con-
dition of the mind takes away all chance
from the body, and often renders him the
victim of an operation, however well pcr-
forine d.?Ru r e uto ike.
M. Sanson has overlooked one very im-
portant cause of the difference of success
in this and other operations, equal in
themselves ?,?namely, the difference of
462 Periscope ; or, Circumspective Review. [Jan. 26
air in a civil hospital, and in a camp, or
military hospital. The two last situations
possess infinite advantages in this respect
over even the best provincial hospitals :
and, if so, how much more salutary must
they be than the smoaky, and malarious
hospitals of a metropolis !
3. HOTEL DIEU DE ROUEN.
Effects of Fear. M. Hellis.
Many curious instances are on record of
the effects (some of them fatal) produced
by sudden emotions of the mind. Barthez
relates the case of a woman, who, having
let fall her infant child, was instantly
stricken with paralysis of one of her arms. ?
M. Hellis lately witnessed a somewhat
similar incident. A young girl, 12 years
of age, having had the curiosity to be
present at an execution,' against the con-
sent of her parents, was so horrified when
the criminal's head was struck off, that
she immediately became paralytic of one
arm. She was under treatment three
months at the Hotel Dieu, without the
least relief, and has since been discharged
uncured. This girl evinced not the slight-
est indisposition, in any other respect,
and M. Hellis thinks that, had she died
immediately after the paralytic attack,
no structural cause of the paralysis would
have been found. We are by no means
So certain of this. He attributes the pro-
duction of paralysis, in such cases, to a
kind of spasm or convulsion?and com-
pares the accident to one of those violent
mental emotions, where the individual's '
voice is instantly taken away, the respi-
ration rendered short, and the heart's
action disturbed. Further, he compares
the paroxysm of terror to a concussion of
the brain, which bereaves the individual
completely of power for the time, or even
destroys life, without leaving any ap-
preciable lesion of structure, on dissec-
tion.
Case 2; A young girl, of nine years
of age> was so frightened by dogs, that
she fell down senseless, and, in this state,
was conveyed, a few hours afterwards, to
the H6tel Dieu. When examined there,
the pulse was calni; the sensibility of
the skin was perfect, the countenance
wits more1 animated than in health, the
eyes wandered without any fixed regard?
all voluntary movement was abolished,
but there was no rigidity, nor did the
members appear to be in a state of com-
mon paralysis. Deglutition was very diffi-
cult. An emetic was administered, and
produced some trifling effect, and, next
day, some leeches were applied to the
neck, without any benefit. On the fourth
day she1 suddenly expired.
Dissection. The dura mater and arach-
noid appeared perfectly healthy. The
brain was carefully sliced, but presented
no alteration or morbid appearance. The
ventricles contained a very small quan-
tity of limpid serum, and their lining
membrane was healthy. The cerebellum
was sound, as were the contents of the
spihal canal. The lungs, the heart, the
liver were healthy. The gall-bladder was
full of green bile. The stomach was un-
altered. The pharynx was the only part
that offered traces of inflammation, and
that to no great extent.?Bibliotheque.
The foregoing is certainly a very strik-
ing instance of the powerful influence of
mind over matter. The longer we live,
the more are we convinced that a great
many disorders and diseases ranged under
the etiology of physical causes in' our
nosological books, are produced by emo-
tions of the mind.
4. HOPITAL COCHIN.
Hydro-pneumo-thorax. Valvular
Disease of the Heart.
The following interesting cases have been
officially published from the CuNiQUEof
the above hospital.
Case 1, (Pneurilo-thorax.) H. Bornier,
aged 22 years, had been affected, fbr a
considerable time, with varices and Vari-
cose ulcers of the legs, and for two years
past he complained of pain in the chest
and difficulty of breathing.' His digestion
was bad?his legs were (Edematous-?he
had very strong palpitations of the heart
?and he could take no exercise without
danger of suffocation. His pulse was full,
hard, and quick. In this state he was
received into hospital, on the 13th June,
1827- He coughed much, and expecto-
rated a yellowish Hocculent matter?com-
plained of deep-seated pain between the
1828] Valvular Disease of the Heart. 463
shoulders. By leechings and low diet
these symptoms were considerably miti-
gated ; but on the 18th fever was re-
kindled, with pain in the right side of
the chest. Leeches, aperients, and di-
luents again employed j but without the
dyspnoea?the muco-purulent expectora-
tion?the quickness of pulse went on ;?
and, or. the 25th, the parietes of the chest
Appeared elevated?little or no respira-
tory sound could he heard in the right
side, which, on percussion, was very so-
norous, and salient. He went on in a
Wretched condition till the 29th, when
he died.
Dissection. A trochar was pushed into
the right side, when a large quantity of
air rushed out. On opening the chest,
the right lung was found compressed
against the vertebral column?its lower
portion and also the diaphragm were co-
vered with false membranes?five ounces
of transparent fluid in that side of the
chest. The fistulous opening in the lung
was found near the summit, and commu-
nicated with a very small cavern in the
superior lobe. Through this fistula the
air escaped and produced the pneumo-
thorax. There were many crude tuber-
cles in the lungs. The mucous membrane
of the trachea was much inflamed, and
covered with muco-purulent secretion.
All the other parts were sound.
Case 2, (Disease of the Heart.) Eliza
Beaugrand, aged 1/ years, of pallid com-
plexion, and delicate constitution, fell
down about two months previously, de-
prived of sense and motion. From that
time, till her entry into hospital, (15th
June,) the extensor muscles of the left
hand remained paralytic, notwithstand-
ing the use of strychnine. On the 17th
the paralysed hand was much infiltrated
?18th, the lower extremities were ob-
served to be oedematous?and, on the
20th, the state of the heart was examined.
She had never experienced palpitation?
the action of the heart was rather strong
?and the whizzing, or bellows noise,
(bruit de soufflet,) was loudly heard by
the ear. On the 22d, when the hand was
placed on the region of the heart, a
trembling sensation (fremissement) was
felt, which it is difficult to describe, but
which can never be forgotten after being
once felt. In the beginning of July, the
dropsical infiltrations, and the bellows
sound increased?and bronchitis was ad-
ded to the other disease. There was
complete orthopncea. She died on the
J 4 th July.
Dissection. On one side of the right
olfactory nerve, there was found a puru-
lent depot, an inch and a half in length,
by three lines in depth, containing white
matter, intermixed with granulations of
more firm consistence. The auriculo ven-
tricular valves of the heart were indurated
?one being of a fibro-cartilaginous tex-
ture, the other partially ossified.?Jouru.
Gen. de Mcdecine.
Remarks. It is very uncommon to find
an apoplectic and paralytic affection in a
person so young, and of such a constitu-
tion as the above female. It is still more
rare to see induration of the mitral and
tricuspid valves at the age of 17 years.
The trembling sensation conveyed to the
hand, in such cases, is very remarkable
?but the noise of the heart's action, as
heard through the stethoscope, or by the
naked ear, is still more striking. We
have compared it, on other occasions, to
the noise made in churning?and, in fact,
the valvular imperfections above des-
cribed, do actually produce a tumultuous
motion of the blood in the different cham-
bers of the heart, resembling the motion
of milk in a churn. At every contraction
of the ventricles a portion of blood is
ejected into the arteries, while another
portion regurgitates into the respective
auricles. This is a terrible disease ; the
sufferings which it occasions in the ani-
mal economy are dreadful; and death is
the greatest blessing that can come on
the wretched patient! These sufferings
are often aggravated by ignorance of
the real nature of the malady?a malady
which we maintain can only be ascer-
tained by proper examination of the chest.
Let those who neglect or obstruct the
study of auscultation, ponder on the res-
ponsibility which they incur by their con-
duct?and let them, moreover, be in-
formed, that all their neglect and ob-
struction of the said study, will have- no
ultimate effect in preventing the culti-
vation of a physical mode of diagnosis,
which is almost the only one, in patho-
logy, that is not imbued with error!
464 Periscope ; or, Circumspective Review. [Jan. 26
,5. HOPITAL ST. LOUIS.
Urticaria Tuberosa Intermittens.
By Dr. Cazenave.
Daubenton, aged 33 years, entered the
St. Louis Hospital, on the 2d December,
1825, and was discharged on the 1st
November, 1826, after a sojourn of 333
days. Four years previously he caught
the itch, of which he was cured by the
usual means. After taking a warm bath,
at the completion of the treatment, he
was seized with a violent rigor succeeded
by intense heat, and ultimately profuse
perspiration. At the same time, the left
elbow and left knee swelled, and pre-
sented a number of irregular nodosities,
of vivid red colour, and accompanied by
insupportable pricking heat. This pa-
roxysm (for so it deserves to be called)
lasted eight hours, and then ceased, leav-
ing the parts above-mentioned covered
with dark-coloured patches, that disap-
peared under pressure. On the succeed-
ing evening, at the same hour, Daubenton
was seized with a similar paroxysm in all
respects, which lasted six hours. The
swellings and nodosities in this paroxysm
were on the opposite side of the body.
From this time, he was regularly seized,
every day, with an attack similar to the
first, except that almost every part of the
body became, in succession, the seat of
the swelling, irritation, and eruption.
Ulcers formed on the ankles, and he was
soon confined to bed, in a most miserable
condition. At the end of a fortnight he
was carried to the H6pital ue Beauvais,
the paroxysms having now augmented in
duration and intensity. The tumefaction
of the lower extremities was now perma-
nent and enormous, the skin being almost
black. Venesection, blisters, sulphure-
ous baths, were employed in this hospital,
by which the complaint was much miti-
gated ; but, as soon as he began to move
about the wards, he relapsed as bad as
ever. After four months residence in
hospital, he went out in the above-men-
tioned condition. He came to Paris, and,
after a few months, repaired to the Ho-
pitai> St. Antoine, where he stayed nine
months, undergoing a variety of treat-
ment, with the effect of reducing the dis-
ease from a quotidian to a tertian, and
sometimes a quartan form. This amelio-
ration was attributable to bark, which he
took in powder. He went out of hos-
pital in this state, and contracted a syphi-
litic complaint, of which he was cured
in the Hopital des Venekiens, the ori-
ginal malady still continuing, though con-
siderably mitigated. The unhappy Dau-
benton now supported a miserable exis-
tence, by working during the early part
of the day, betaking himself to bed every
evening, under a paroxysm of the pristine
affection. At length he presented himself
at the St. Louis, and there the periodical
accessions of the disease, as already des-
cribed, were unequivocally ascertained.
The paroxysms generally lasted four or
five hours, accompanied by the swelling _
and eruption above-mentioned. On mi-
nute examination, during the remissions,
no organ of the body appeared to be dis-
ordered in function or structure. Having
now again become affected with itch, he
went through the usual regime for that
malady, and was cured of it. The sul-
phate of quinine was then administered,
in doses of eight grains per diem. The
paroxysms were stopped by this remedy,
and he remained eight days free from
complaint. He procured wine secretly
?committed a debauch?and again the
original malady returned. This relapse
was accompanied by inflammatory symp-
toms, which obliged them to omit the
quinine, and prescribe antiphlogistics for
a fortnight. Again the quinine was ad-
ministered, and, in twelve days, the pa-
roxysms were stopped. The intermission
lasted a month, when the disease re-
turned, without any appreciable cause j
but in a much milder form. M. Biett
now determined to employ arsenic ; and
Fowler's solution was administered, at
first in doses of five drops per diem.
The paroxysms ceased after the fifth day
of this treatment. The remedy was ob-
liged to be suspended for eleven days, on
account of some enteric affection, but the
original malady did not appear in that
interval. The solution was then recom-
menced, and continued for 26 days, at
six drops the dose. The malady returned
no more, and the patient was retained
six weeks in the hospital, after cure, in
order to be sure that he was free from
complaint.?Bibmotheque.
The above we conceive to be a very
interesting case, and certainly it does
credit to the arsenic, which, in this, as
in many other periodical affections, is
even superior to the quinine,
1828] Fractured Ribs, &c. 405
6. ROYAL INFIRMARY OF
EDINBURGH.
Fractured Ribs.
Some time ago, a person, who stiled him-
self Erinensis, took to writing letters in
the Lancet, which, though frothy and su-
perficial enough, still had a degree of
talent and wit in them, and might have
made excellent briefs for a second-rate
Irish barrister at the Old Bailey. Such,
however, was the success of this gentle-
man from Dublin, that Edinburgh, deter-
mined not to be behind-hand, sent forth
her champion under the designation of
Scotus, who followed his predecessor
" haud passibus eequis." Whether the
Reports of Scotus be true or false we
cannot pretend to say; if, however, they
are the latter, there are plenty of chan-
nels now open to the medical officers of
of the Royal Infirmary to expose their
misrepresentations, and it is their own
fault if they do not take advantage of
them. In No. 228 of the Lancet the fol-
lowing case is related.
W. G. set. 68, broke his 6th and 7th
libs, near the angles, by a fall on the
25th September last. He applied no ban-
dage, and walked about as usual until the
3d October, when he entered the infir-
mary, under the care of Dr. Hunter.
He had then severe pain in the side, dysp-
noea, pulse 100, bowels costive, tongue
white. V.S. ad |xxx. with relief. On the
next day the stethoscope indicated "an
alteration in the pulmonary tissue" in
the situation of the fracture. On the 6th
he was bled to ?xvj. and this not giving
relief, a second bleeding was practised,
after which he went on well until the
11th, when he had a rigor in the night,
and inflammation took place in the vein
which had been opened. Incisions were
made into the arm, as it was generally
swollen, and it was enveloped in ice, by
which means, aided by bleeding and sa-
lines with antimony, the pain and swell-
ing of the limb gradually subsided, and
little remained except a short cough, and
pain in the side. On the 21st, he had
severe dyspnoea, which was relieved by
a blister, but returned, on the 23d, with
excessive pain in the neighbourhood of
the fractured ribs. On examination, there
was found an inflamed, circumscribed,
and fluctuating tumour, reducible by pres-
sure and position apparently into the ca-
vity of the cliest. On the 4th November
Dr. Hunter, after a short consultation
with his colleagues, made an opening into
the pleura, between the 6th and 7th ribs,
where the tumour was most prominent,
and gave issue to a " few ounces" of
healthy purulent matter. The Doctor,
we are informed, passed his fingers be-
tween the fractured ends of the ribs, and
distinctly felt their rough extremities, no
attempt at union having taken place. A
piece of oiled lint was introduced into the
wound, the discharge from which pro-
gressively diminished, until the 25th of
December, when it had entirely cica-
trized, and the patient is to leave the
hospital in a few days.
We agree with the reporter, that this
is not to be considered as a case of em-
pyema, or as in any degree affecting the
question of paracentesis thoracis in that
formidable disease. We are not quite
satisfied, however, that the formation of
matter, in this case, depended exclusively
on the fractured ribs, for, if so, it ought
to have given note of its existence sooner.
It evidently shewed itself subsequently to
the inflammation of the veins of the arm,
and every one who has seen many of these
cases of phlebitis, after bleeding, must be
aware, that inflammation and the depo-
sition of matter in the pleural cavity is
by no means an unfrequent result. We
do not mean to deny, that the fracture
may have had a share in the production
of the disease, but we certainly are of
opinion, that the affection of the veins
had also a share, and that a considerable
one, in its development.
7. MEATH HOSPITAL, DUBLIN.
Dks. Graves and Stokes on Cuta-
neous Diseases.
Every ofie knows the difficulty of curing
cutaneous diseases ; which is about as
great as the anxiety of patients to have
cures speedily effected. Accordingly
skin-doctors are in as great demand as
spine-doctors?sometimes more so. It
is wonderful how much may be done by
the milliner in concealing a distortion ;
but an unsightly eruption on the face,
the os sublime by which Heaven is said
to have distinguished man from all other
animals, though, in reality, a monkey's
466 Periscope; oh, Ciucumspective Review. [Jan. 26
face looks more upivards than that of the
philosopher, is a terrible defect, and the
removal of it is eagerly sought, even at
the expense of some more important
malady.
We are informed by the physicians
above-mentioned that, at the date of their
reports, they had, in the Meath Hospital,
a young girl, labouring under the severest
form of psoriasis, affecting not only the
scalp, face, and extremities, but almost
the whole surface of the body. A most
abundant desquamation of silvery white
scales was constantly taking place, so
that handfuls of them could be ga-
thered in her bed every morning. Her
skin was almost universally of a bright
red colour, and was very itchy?pulse
100, and strong?thirst?but otherwise
healthy. She was twice bled?put upon
low diet?and leeches were repeatedly
applied to the most inflamed parts of the
surface. In the mean time, she took
daily, a pint of decoctum sarsae, with
two drachms of supcrtartrate of potash.
When the inflammatory symptoms had
subsided, sulphur was exhibited inter-
nally, with warm baths containing sul-
phuret of potash. The disease dimi-
nished for some time, and then made a
stand against the doctors. Jar-ointment,
and afterwards the ung. hydrarg. nitrat.
were employed, with good cleansing of
the surface, and sulphur internally con-
tinued, by which means a complete cure
was ultimately effected.
" In squamous diseases, more limited
in their extent, we have successfully used
a similar method of treatment; except
that in such cases general bloodletting
may be often dispensed with, as the re-
peated application of leeches to the
affected parts is sufficient to subdue the
active inflammatory stage of the disease.
The only difficulty which occurs in the
treatment of squamous disease^, is to
determine the proper period for leaving
off the local antiphlogistic applications,
and changing them for stimulants. The
latter, if applied too soon, will aggravate
the disease, and when this is found to be
the case, they should immediately be laid
aside, and the application of leeches,
poultices, and cooling lotions again be
resorted to."
Hitherto Drs. Graves and Stokes have
met with no case of scaly disease,
which has resisted this simple method
of cure. They have also extended these
principles to the treatment of chronic
pustular and tubercular diseases, and
with considerable success. A similar
treatment they have found efficacious in
a case of sycosis menti?and they have
used it, with marked advantage, in por-
rigo of the scalp, or tinea capitis. Re-
cent cases of porrigo, they say, yield
readily to the application of leeches to
the head, with poultices repeated till the
inflammation of the scalp is subdued.
This may be known by the decrement
or disappearance of the heat, redness, and
soreness of the scalp. As the formation
of pustules depends on the inflammation,
their development will cease with the re-
moval of the cause ; and the cure may
then be readily effected by the use of the
tar and citrine ointment. It is hardly
necessary to remark, that the prelimi-
nary steps of lulling the pain, shaving
the head and applying poultices, alkaline
solutions, &c. till the scabs are softened
and removed, are necessary. Even in
very chronic cases, they have found it
best to commence by leeching the head ;
but recent cases require, of course,
a more antiphlogistic treatment than
chronic.
In many cases of scald head, where
ciix'umstances have prevented leeching
in the first instance, poultices, with an
ounce of the liq. acet. plumbi, have been
substituted with advantage. In children,
this will often succeed in x-educing high
inflammatory action. In one case, how-
ever, the continued application of the
acetate of lead produced something like
colica pictonum. The following remark
is worthy of attention. " There can be
no doubt that the sudden drying up of
cutaneous diseases has occasionally pro-
duced dangerous internal complaints.
This danger does not seem to attend their
cure by the antiphlogistic treatment,
which, when prudently conducted, dimi-
nishes the tendency to inflammatory
action in the constitution, and does not,
like merely topical applications, destroy
it in one part, only to re-appear in
another."
1828] Transactions of Societies. 4G7
TRANSACTIONS OF SOCIETIES.
1. Aneurism.
a late sitting of the Westminster Medi-
cal Society, Dr. Barry furnished a subject
for discussion?namely, that of internal
aneurism. The reason for the internal
consideration of the subject was humour-
?usly enough grounded on the distinction
between physic and surgery. Thvis, till
an aneurism points above the clavicle or
below Poupart's ligament, it is, of course,
the property of the physician?but the
moment it overleaps these boundaries, the
surgeon claims it as his prescriptive right!
True it is, that the surgeons sometimes in-
vade the domain of the physician. Cooper
and Abernethy ought to be prosecuted
and fined by the College of Physicians
for digging into the abdomen?a soil in
which they had no right to plunge a
spade ; while Wardrop and many others
have passed the line of demarcation, in
an opposite direction, and have theatened
to tie up the ventricles or auricles of the
heart itself, when affected with aneurism !
We have a plea for canvassing the opi-
nions and practices of both parties?be-
pause, like Tyresias, of old, we have served
*n both capacities.
If aneurism were as common in ancient
as in modern times, it is wonderful that
it should have escaped the observation
?f Hippocrates and Celsus. The latter
clearly understood the difference between
veins and arteries, and observed that
Wounds of the latter would not heal.
" At arteria incisa neque coit, neque
sanescit ; interdum etiam, ut sanguis
vehementer erumpat, efficet."?Lib. ii.
&ut the word aneurism does not occur in
his writings. He describes varices of the
veins of the leg, and directs them to be
cut out, having first placed a ligature
above and below the tumour. In wounds
of the veins, too, he directs a ligature
above and one below the wound, and
then the vessel to be entirely cut across.
" Vencc, qute sanguinem fundunt appre-
hendendae, circaque id quod ictum est,
duobuslocis delegandse, intercedendseque
sunt."?Lib. v. It must not be said that
Celsus here means an artery when he
speaks of a vein ; for he has minutely
described the differences between these
vessels, not only in their coats, but in
the kinds of blood which they contain.
If this opinion be doubted, we have the
authority of Sprengel on our side.
^Etius seems to be the first who dis-
tinctly notices aneurism, when speaking
of Philagrius. He says that that bold
snrgeon excised the aneurismal tumour
in toto, having first tied the artery above
and below. Paulus Eginetus, and almost
all the surgeons of the middle ages, follow-
ed the above method; which afterwards
fell into disuse. Lanfranc was the first
to recommend the actual cautery to the
aneurismal opening in the artery, which
advice was adopted by Fallopius. Marcus
Aurelius Severinus appears to have been
the first who tied the femoral artery
close to Poupart's ligament, for an aneu-
rism at the top of the thigh.* Thus, till
the sixteenth century, the treatment of
aneurism was by ligature?and, in some
rare cases, by excision of the tumour.
At this epoch, John of Vigo tried com-
pression and styptics, which were em-
ployed by Ambrose Pare, Fabricius
Hildanus, See. About the middle of the
seventeenth century, the tourniquet was
employed by some surgeons, to check
the flow of blood into the aneurismal sac,
while various contrivances were adopted
to make pressure on the tumour itself.
Passing over these, it appears that
Dominique Anel was the first to propose,
and Lacorta to perform the operation of
tying the artery both above and below
the aneurism, without making any open-
ing into the latter. Then came the ope-
ration of John Hunter, so Avell known,
and still so universally adopted. But we
cannot always, as in popliteal aneurism,
tie the artery at a distance above the
disease?nay, we cannot always get at
the artery at all, between the aneurism
and the heart. This consideration" led
Dessault to propose, and it is said (by the
younger Sprengel) to perforin, with suc-
cess, the ligature beyond the tumour.
This operation is reported to have suc-
ceeded in aneurisms of the axillary and
external iliac arteries, but we cannot
find the proofs. Deschabips followed
the example of Dessault; but in the
two operations which he performed for
aneurism of the external iliac or inguinal
* De EfficaciMed. Lib. i. p. 11. p. 51.
46S Periscope; on, Circumspective Review. [Jan. 26
artery, he completely failed. The same
want of success attended Sir Astley
Cooper, and it only remains to. inquire,
whether those performed by Mr. Ward-
rop, on the principle of Dessault, promise
a more fortunate result. Of the four
patients operated on by Mr. Wardrop
and Mr. Lambert, two are dead, and in
one of these, no aneurism, it has been
shewn, was found, nor any trace of li-
gature having been applied to the caro-
tid artery. The first case, the old lady,
is still living, and dissection has not yet
cleared up the true nature of the malady.
Of the case operated on by Mr. Lambert,
we gave our opinion in a former Number.
From seeing the preparation and the
drawing, we are still of opinion, that it
was aneurism by dilatation of the carotid
artery, a disease not necessarily fatal in
itself. The patient died of haemorrhage,
from ulceration of the coats of the artery.
Of the last case operated on by Mr.
Wardrop, we gave an account in our last
Number, page 534. Dr. Barry's diag-
nosis of the disease may be seen in the
same place, where he stated the innomi-
nata to be considerably dilated, as also
the aorta within the pericardium and at
its arch, where it pressed upon some of
the bronchia. Dr. Barry also stated his
opinion, that the left carotid was " uni-
formly-dilated." From these and other
considerations, Dr. Barry pronounced the
disease to be fatal in its results, and that
110 operation on the right carotid could
be of any service. Nevertheless the sub-
clavian was tied, and in the Lancet, we
find the most flourishing accounts of the
patient's subsequent health. She had
lost her cough, her dyspnoea, all pain?
and could go up stairs as well as ever.
But Dr. Barry, in the Westminster
Society, stated, from an accurate exami-
nation of the woman, that the dyspnoea,
instead of being removed, was increased
since the operation?and in short, there
cannot be a doubt that, although the
tumour or dilatation of the vessels was
prevented from proceeding in an outward
direction, it had been making progress in
another and more dangerous direction,
which would terminate ultimately (if it
went on) by suffocation. Thus the pa-
tient had been relieved of pain, occa-
sioned probably by the pressure of the
aneurism on some nerve or nerves, but
had got an increase of dyspnvca, which
we consider a far more formidable symp-
tom, and one which was likely to pro-
duce more suffering in the end.
From this expose, we believe the Pro-
fession will be disposed to place little
hope in the ligature ultra tumorem.
When, indeed, an internal aneurism has
protruded beyond the clavicle or the
groin, there can be little rational hope of
cure by any means. The cause of want
of success, may fairly be attributed to the
impossibility of arresting the current of
blood through the aneurism, on which ar-
rest the great hope of success must depend.
Now the epigastric and circumflexa ilii
arteries must always keep up a stream
through an aneurism, where the ligature
is applied beyond the tumour, in the
groin or thigh?and the carotid, and
several other arteries must also keep up
the current in an aneurism of the inno-
minata ; so that the chance of success is
diminished almost to a cipher. The only
tangible artery, as we stated in a former
Number, where ligature ultra tumorem
offers any prospect of cure, is the carotid,
because, from the common trunk of that
vessel, no branch goes off till it divides
at the top of the neck. In an aneurism,
therefore, of that artery, situated too low
for ligature citra aneurismam, there is
a fair excuse for tying the vessel ultra
tumorem. In any other situation, wc
fear the operation will be useless?per-
haps, worse than useless.
P. S. Just as we had penned the above,
Mr. Wardrop's additional report, dated
the 5th December, came to our view. In
this communication Mr. Wardrop im-
pugns the statement made by Dr. Barry,
above alluded to, and he tells us that at
the end of August, Mrs. Denmark was so
well that Mr. Chapman, who examined
her, declared to Mr. Lawrence that, had
he not been previously acquainted with
the nature of the case, " he could never
have discovered that aneurism of any of
the large vessels of the heart had ever ex-
isted." Lancet, Dec. 8. Mr. W. further
informs us that the patient's countenance
had "lost entirely that anxious expres-
sive look which was formerly so observ-
able." And bringing up the account
to the date of his report, (5th Dec.) says,
" she has regained a nearly perfect state
of health." The operator avers that, six
weeks prior to the above date of report,
1828]
Transactions of Societies. 469
" not a vestige of the tumour remains "
perfect cure liad been effected."
Such was the statement on the 5th De-
cember. On the 12th December, we ex-
amined Mrs. Denmark with the greatest
pare. Her countenance was the very
image and personification of distress and
despair. Her breathing was extremely dif-
ficult?not the dyspnoea of inflamed lungs,
but the difficulty of breathing through a
compressed tube. The noise made by the
e'fort to fill and empty the chest, was
Precisely that made by the rush of air
through a flattened tube. Examination,
by the stethoscope and pleximeter shewed
the existence of the original aneurismal
tumour, stretching from the heart to-
wards the first lib. The pulse and im-
pulse of the tumour were still unequivo-
cal. It was perfectly evident that the
tumour was pressing on the air-passages,
and was thus the cause of the dreadful
dyspnoea, as it would be, ultimately, of
the death of the patient. Such was the
state of this unfortunate woman's case,
on the 12th December, four days after
Mr. Wardrop's statement appeared before
the public. We cannot reconcile the dis-
crepancy of Mr. Wardrop's statement
with the evidence of our own senses. We
again saw this woman on the 16th De-
cember. She was then confined to her
bed, and obliged to keep the trunk nearly
perpendicular. The breathing was still
more difficult than before?and a muco-
purulent expectoration was brought up
with difficulty from the lungs. On this,
and on the former visit, we found the
pulse in the right arm perfectly distinct,
though not so strong as in the other arm.
We have been informed, on the very best
authority, that on the very day after the
operation, and afterwards on the 10th or
12th day, consequently before the liga-
ture came away, the pulse was distinctly
felt by Mr. Lawrence and others, in the
right arm. If this information be incor-
rect, Mr. Lawrence can easily contradict
the statement, when we will give our au-
thority, who also felt the pulse.?Was the
artery tied?or, at least, was the canal
obliterated ?
On the 2/th December, we again saw
Mrs. D. Bleeding and leeching had oc-
casionally relieved the difficulty of breath-
ing ; but the pressure of an aneurismal
tumour on the trachea or bronchia was
fearfully evident. At this time, we could
not perceive the beat of the radial ar-
tery. The pulsation in the left carotid
was tremendous. A kind of undulatory
pulsation was perceptible in the situation
of the original aneurism ; but whether it
was a communicated pulsation, or a motion
of blood in the part, we could not dis-
tinctly ascertain. A muco-purulent ex-
pectoration was still thrown up?and
when the expectoration accumulated, the
difficulty of breathing was dreadful. The
unfortunate patient could only repose in
a nearly erect posture, and her sufferings
were extremely distressing.
On Saturday, the 5th January, we had
reason to know (though not from personal
observation) that the aneurismal tumour
was again taking an outward direction?
that the clavicle was again rising, and
being dislocated?and that, in short, the
poor patient was nearly in the same pa-
thological condition as before the opera-
tion of tying the subclavian artery?ultra
aneurismam ! This direction of the aneu-
rismal tumour outwards, and the repeated
bleeding which had been practised, had
somewhat relieved the dyspnoea, and the
muco-purulent expectoration was much
lessened.
Our information respecting this poor
woman comes down no later than the 14th
January, when we understood that the
clavicle was still rising?that the breath-
ing was still very bad, though not so
much so as at one or two periods within
the last six weeks?and that no pulsation
whatever could be felt in the right arm
since the 2/th December.
Now, as we can confidently state that
the pulse was felt in the right wrist the
day after the operation, and at all times
up to the 2/th December?and as all
pulsation has now ceased in that arm, are
we not authorised to suspect that the sub-
clavian was never tied*?and are we not
fully justified in asserting that, the cessa-
tion of pulsation in' the arteries of the
arm, of late, is solely owing to some pres-
sure, or morbid change going on in the
* We are aware that it is not very
uncommon for the pulse to return in an
artery, after the Hunterian operation has
been performed. This return, however,
is only temporary ; the pulsation is more
or less indistinct, lasts for a few days,
and disappears.
470 Periscope ; or, Circumspective Review. [Jan. 26
chest, and totally unconnected with the
operation ?
Upon the whole, a careful examination
of all the cases now on record, including
that of Mrs. Denmark, induces us to con-
clude that the ligature ultra aneurismam
has failed, and that there are no facts to
authorise a repetition of it. We conceive
that we should not have been acting con-
scientiously to the profession, and to hu-
manity, had we suppressed the true state
of Mrs. Denmark's case, after a reporthad
gone forth that she was perfectly cured.
2. Arm and Shoulder-presentations.
Dr. Robert Lee read a short paper before
the Westminster Medical Society, on the
5th January, the object of which was to
recommend the sacrifice of the child, in
cases of arm and shoulder presentations,
where turning was unadvisable or im-
practicable. He shewed that much mis-
chief to the mother is sometimes done by
attempts to turn, without saving the child
after all. When, therefore, we have proof
that the child is dead?that the pelvis is
too small to allow a living child to pass
?or, that there is great improbability of
bringing a living child into the world,
even by turning, we are advised to dis-
joint the arm at the shoulder, introduce
a blunt hook, so as to take hold of the
spine, as low down as possible?and bring
away the foetus double?thus imitating
the operations of nature, in some cases
of spontaneous evolution. Dr. Lee did
not bring forward this procedure as en-
tirely new, since Dr. Douglas and some
others had proposed it. He related three
cases in illustration. Some comments
were made by Mr. Jewel, as to the no-
velty of the proposal, but no serious ob-
jections made to the practice.
3. Purulent Depots, after Wounds
and Operations.
Mr. Rose, of St. George's Hospital, read
a paper to the Medico-Chirurgical So-
ciety, on the 7th January, on the above
subject. It has been observed by various
foreign and English writers, that abscesses
(as they were called) occasionally took
place in the liver and lungs, especially
in the former viscus, after wounds, ope-
rations, and accidents. According to
continental experience, the liver is the
organ most commonly affected ; but, in
English practice,, the lungs are full as
often the scene of the purulent deposi-
tion. The peculiarity is, that the matter,
if it be entitled to that name, is collected
in one or more globular depots, not at
all like common abscesses of the parts,
the surrounding structure being generally
sound. The deposition itself is a mixture
of coagulable lymph'and puriform striae.
Mr. Lawrence related a case, where the
surface, and even the substance of the
liver, were found studded with some hun-
dreds of these globular depots, after an
operation on the knee-joint. A warm
discussion took place between Dr. Sey-
mour, Mr. Lloyd, and Dr. Johnson, as to
the frequency or infrequency of common
circumscribed abscess (unconnected with
tubercles) in the parenchymatous struc-
ture of the lungs, in acute inflammation.
According to Dr. Seymour, the occur-
rence was by no means uncommon?ac-
cording to Mr. Lloyd, one would suppose
that, nothing was more common?accord-
ing to Dr. Johnson, it was extremely
uncommon?so much so, that he had
never met with a well-marked instance
of it, as proved by dissection.* The au-
thority of Laennec was opposed by that
of Dr. Luke! This put an end to the
discussion. As Mr. Rose's paper will
soon be published, we shall abstain from
any remarks at present; but we promise
to meet Messrs. Seymour and Lloyd be-
fore a larger audience, on the above-
mentioned subject, ere long.
4. Auscultation.
In two recent sittings of the Westminster
Medical Society, the important subjects
of auscultation and percussion were warm-
ly discussed; and never was triumph more
complete than that obtained by the advo-
cates of these modern means of ascertain-
ing thoracic diseases! In compassion to
the opposers of these improvements in
medical science, we shall draw a veil
over their names. There were only three,
* Mr. Lawrence only remembered one
example, and that appeared such a rarity,
that he preserved the part in his museum.
1828]
Transactions of Societies. 471
j11 a society consisting of nearly 100 mem-
bers and visitors. On the first night,
there was a diarrhoea, we might say a lien-
tery of declamation poured forth against
auscultation and percussion, without a
single fact or argument being adduced,
that was worthy of refutation. Two facts
only were brought forward, and these we
shall state, without deigning to honour
them with a comment. The first fact
occurred before auscultation was born.
A young woman was admitted into the
Royal Infirmary of Edinburgh, some 20
?r 30 years ago, with swellings in both
htiees. The surgeons, anxious to shew
their dexterity, amputated one of the
lower extremities; and, on examining the
joint, found there was no organic disease
to authorise the operation. They then
set their brains to work, and cured the
disease in the other knee, without ampu-
tation :?ergo, auscultation was a most
pernicious measure introduced into medi-
cal diagnosis ! As our country readers
might not believe that such a fact could
be seriously presented to a medical so-
ciety, as an objection to auscultation, we
shall explain a little. The gentleman
who introduced this case, in the way of
illustration, observed that, as the Edin-
burgh surgeons were more intent on
shewing their dexterity with the knife,
than their therapeutical knowledge in
the cure of the swelling of the knee, so
the auscultators would neglect all inves-
tigations, except by the stethoscope, and
thus become non-observers of the pheno-
mena of thoracic diseases !!
Fact the Second. Two patients died in
the Middlesex Hospital, on one of whom,
a student had been making some ste-
thoscopical observations. On repairing
to the dead-house, the young pupil began
to examine the wrong body, through a
mistake?ergo, the dreadful injuries which
must result to medical science from this
zeal which some young and raw students
evince in opening bodies (wrong bodies)
after death !
These were the only facts brought into
court against auscultation and percus-
sion ; and the anti-auscultators candidly
acknowledged, that they had never made
the least attempt to examine into the
value of the diagnostic measure ! They
poured forth an abundant declamation
against the stethoscope, as a " foreign
bauble," an "inutile lignum"?an " in-
suit to John Bull"?and, in short, a piece
of " continental quackery." They had
(like Mr. Lawrence) the last tvords, in
the evening of the first night. But a
terrible humiliation was in reserve for
them in the second night's discussion.
On this night two out of the three ratted,
and passed the highest eulogia on auscul-
tation ; while the third was. completely
nonplused, and merely entered his protest
against auscultation, without adducing a
single fact or cogent argument against
the measure.
On the other hand, the value and uti-
lity of this auxiliary to diagnosis was ably
maintained by Mr. Mackelcan, Dr. Barry,
, Dr. Shiel, Mr. Bennett, Dr. Milligan,
Dr. Johnson, and others. Dr. Barry, in
a luminous speech, shewed the wretched
state in which the pathology of the chest
was, before the days of Avenbrugger,
Corvisart, and Laennec. It is true that
Dr. Baillie has left us a description of
diseased appearances, which is fit for
nothing, except a catalogue of a museum.
Dr. B. described the morbid changes be-
fore he saw the symptoms?and when
he described the symptoms, he had no
opportunity of seeing the morbid changes.
Thus, the connexion between symptoms
and changes of structure, in Dr. Baillie's
work, is purely imaginary. Yet, on this
connexion, the whole value of pathology
depends. Hence the great merit of Laen-
nec and other modern pathologists con-
sists in demonstrating tiie external signs
of internal morbid processes.
Among other futile and erroneous ob-
jections that were made to auscultation,
it was urged that, when diseases were so
far advanced as to be recognizable by the
stethoscope, they were incurable?ergo,
auscultation was useless. A damper was
thrown on this line of argument by the
case of a man, residing within 500 yards
of the Society's rooms, and who had been
seen by three physicians then present in
the Society. This man had been in two
public institutions?in one he was treated
for nervous asthma?and ordered to take
beef-steaks and porter. There he got
worse ; so he went to another institution,
where he was treated for phthisis?and
there he got no better. Dr. Johnson was
requested to see this poor man, and, on
examining the chest, an aneurism was
discovered, without the least difficulty,
beating under the right clavicle. The
472 Periscope; ok, Circumspective Review. [Jan. 26
case was shewn to Dr. Barry and another
physician, who were appealed to for the
accuracy of the statement. The man was
bled, put upon low diet, and his breathing
was, by these means, much relieved.
Here, then, was an instance of the inju-
rious consequences of auscultation. Dr.
Barry related two or three recent cases,
where the most erroneous views of dis-
ease had been taken, and where an in-
jui'ious mode of treatment was being
pursued, till auscultation set the practi-
tioners on the proper road. In fine, the
the anti-auscultators were reduced to a
single theme of declamation?namely, that
the stethoscope would render the rising
generation of the profession less attentive
observers than their fathers and grand-
fathers. We have only to say, that it
will have just the opposite effect. It
will make them much better observers.
Ligature of the Common Iliac
Artery. Dr. V. Mott.
This operation, which throws into the
shade every other of the kind, yet placed
on record, has recently been performed,
iv'dh success, by one of our transatlantic
brethren. We shall proceed, at once, to
detail this brilliant triumph of modern
surgery.
On thel5th March, 1827, Dr. Mott was
called, with Dr. Osborn, to a patient, who
was foundlabouringunderalargeaneurism
of the right external iliac artery. The
man's name was Israel Crane, aged 33
years, a farmer, of temperate habits, but
accustomed to hard and laborious work.
About the middle of January he felt pain
in the lower part of the belly, which he
attributed to a fall formerly received. It
was only a fortnight previously, however,
that he perceived the tumour. On exa-
mination, the abdomen, on the right side,
was considerably enlarged, from the cru-
ral arch to the umbilicus, the swelling
being pulsatile, both to the touch and the
sight. It appeared to contain fluid blood
only. It commenced a little above Pou-
part's ligament, reached nearly to the
navel, advanced forward near the linea
alba, and seemed to fill up all the conca-
vity of the ilium.
" The rapid increase of this aneurismal
tumour occasioned, as the. countenance
of our patient indicated, the most extreme
agony. His sufferings at times were so
great that his screams could be heard at
a distance from the house. He had been
bled several times, taken light food, and
was kept constantly under the effect of
opium. He was now informed of the se-
rious nature of his case, and that without
an operation, very little chance of his life
remained ; with great composure he im-
mediately consented to whatever would
give him the best prospect of saving his
life.
" From the extent and situation of the
tumour, he was apprised of the uncertain
nature of the operation, as well as the
difficulty of performing it, and indeed that
it would require an artery to be tied,
which never had been before operated
upon for aneurism. With these views of
his situation, he cheerfully submitted to
be placed upon a table of suitable height
in a room which was well lighted.
" Then, in the presence of Dr. Osuokn,
Dr. Liddle, and Dr. Cross, the follow-
ing operation was performed :?
"The pubes and groin of the right side
being shaved, an incision was commenc-
ed just above the external abdominal ring,
and carried, in a semicircular direction,
half an inch above Poupart's ligament,
until it terminated a little beyond the an-
terior spinous process of the ilium, mak-
ing it in extent about five inches The
integuments and superficial fascia were
now divided, which exposed the tendinous
part of the external oblique muscle, upon
cutting which in the whole course of the
incision, the muscular fibres of the inter-
nal oblique were exposed; the fibres of
which were cautiously raised with the
forceps and cut from the upper edge of
Poupart's ligament. This exposed the
spermatic cord, the cellular covering of
which was now raised with the forceps,
and divided to an extent sufficient to admit
the forefinger of the left hand to passupon
the cord into the internal abdominal ring.
The finger serving now as a director,
enabled me to divide the internal oblique
and transversalis muscles to the extent of
the external incision, while it protected
the peritoneum. In the division of the
last mentioned muscles outwardly, the
circumflexa ilii artery was cut through,
and it yielded for a few minutes a smart
bleeding. This, with a smaller artery up-
on the surface of the internal oblique mus-
1828] Ligature of the Common Iliac Artery. 473
p'e, between the rings, and one In the
"'teguments were all that required liga-
tures.
" With the tumour beating furiously
Underneath, I now attempted to raise the
peritoneum from it, which we found dif-
ficult and dangerous, as it was adherent
to it in every direction. By degrees we
separated it with great caution from the
sneurismal tumour, which had now bulged
UP very much into the incision. But we
s?on found that the external incision did
not enable us to arrive to more than half
the extent of the tumourupwards. It was
therefore extended upwards and back-
wards about half an inch within the ilium,
to the distance of three inches, making
a wound in all about eight inches in
length.
" The separation of the peritoneum
Was now continued, until the fingers ar-
rived at the upper part of the tumour,
Which was found to terminate at the go-
ing oif of the internal iliac artery. The
common iliac was next examined by pass-
ing the fingers upon the promontory of
the sacrum, and to the touch appearing
to be sound, we determined to place our
ligature upon it about half way between
tlie aneurism and the aorta, with a view
to allow length of vessel enough on each
side of it to be united by the adhesive
process.
" The great current of blood through
the aorta made it necessary to allow as
much of the primitive iliac to remain be-
tween it and the ligature as possible, and
the probable disease of the artery higher
than the aneurism, required that it should
not be too low down. The depth of this
wound, the size of the aneurism, and the
pressure of the intestines downwards by
the efforts to bear pain, made it almost
impossible to see the vessel we wished to
tie. By the aid of curved spatulas, such
as I used in my operation upon the inno-
minata, together with a thin smooth piece
of board, about three inches wide, pre-
pared at the time, we succeeded in keep-
ing up the peritoneal mass, and getting
a distinct view of the arteria iliaca com-
munis, on the side of the sacro vertebral
promontory. This required great effort
on our part, and could only be continued
for a few seconds. The difficulty was
greatly augmented by the elevation of the
aneurismal tumour, and the interception
it gave to the admission of light.
" When we elevated the pelvis, the tu-
mour obstructed our sight; when we de-
pressed it, the crowding down of the in-
testines presented another difficulty. In
this part of the operation I was greatly
assisted by Dr. Osborn, and my enterpris-
ing pupil, Adrian A. Kissam.
" Introducing my right hand now be-
hind the peritoneum, the artery was de-
nuded with the nail of the forefinger, and
the needle conveying the ligature was
introduced from within outwards, guided
by the forefinger of the left hand in order
to avoid injuring the vein. The ligature
was very readily passed underneath the
artery, but considerable difficulty was ex-
perienced in hooking the eye of the nee-
dle, from the great depth of the wound
and the impossibility of seeing it. The
distance of the artery from the wound
was the whole length of my aneurismal
needle.
" After drawing the ligature under the
artery, we succeeded by the aid of out-
spatulas and board in getting a fair view
of it, and were satisfied that it was fairly
under the primitive iliac, a little below
the bifurcation of the aorta. It was now
tied?the knots were readily conveyed up
to the artery by the forefingers?all pul-
sation in the tumour instantly ceased.
The ligature upon the artery was very
little below a point opposite the umbi-
licus.
" The wound was now dressed with five
interrupted sutures, passing them not
only through the integuments, but the
fibres of the cut muscles, so as to bring
their divided edges together at all parts
of the incision, which was muscular. Ad-
hesive plaster to assist the stitches, lint
and straps to retain it, completed the
dressing. The operation lasted rather
less than one hour."
The space which we have dedicated to
the steps of the operation, prevents us
from giving a detail of the diurnal re-
ports, carefully drawn up by Dr. Osborn,
and authenticated in the most satisfactory
manner. There was very little constitu-
tional disturbance produced by this for-
midable operation. The temperature of
the limb was soon restored:?The patient
was bled a few times?kept quiet and
low. The operation was performed on
the 15th March, and on the 3rd of April
Dr. Proudfoot removed the large ligature,
the wound being almost healed. On the
Vol. VIII. No. 16. 3 G
474 Periscope; or, Circumspective Review. [Jan. 26
16th the patient imprudently rode out,
without permission, the wound being
perfectly healed. On the 30th April, he
was in complete health. The leg was
not of its full size, nor so strong as the
other?he was free from pain. On the
20th May, he came 25 miles to shew
himself in New York. The remains of
the aneurismal tumour had disappeared
?the epigastric artery was found much
enlarged, and beating strongly, and there
was a feeble pulsation in the femoral
artery.
When we compare this operation with
the one performed some months ago by
Mr. Wardrop, and reflect that in a case
like Crane's, the operation ultra cincuris-
mam would have been applicable, accord-
ing to the doctrines recently maintained,
we must admit that the interests of sur-
gery and of humanity demanded a full
and speedy exposition of the newly re-
vived doctrine of Dessault, and the data
on which it is grounded. The facts
which we have here put upon record
speak for themselves ; and we shall not
add a single comment.
METROPOLITAN HOSPITAL PRACTICE.
1. COLDSTREAM GUARDS HOSPITAL.
Disease of the Heart.
We shall present to our readers a short
statement of this case (from the Medical
Gazette) before we make any comment.
A young soldier (aged 23) was received
into hospital on the l/th of November,
for feverishness and constipation, which
seemed to give way in three days. On
the 26th he had a fresh attack. On the
1st of December Mr. Maynard saw him.
" He appeared very feverish, anxious, and
irritable; had a pulse of very unusual and
remarkable strength ; it was not only visi-
ble at the wrist, but in the Jlesliy parts of
the thighs and belly; his skin hot, with
great thirst." He was bled to ten ounces
from the arm, without any relief, or any
sensible effect on the pulse. From this
time till the 10th December he varied
much; but, at the latter period, com-
plained of pains in his knees, breast,
shoulders, and bowels. On the 23d the
fever was renewed, and calomel and opium
were given at night, without benefit. A
troublesome cough was now added to the
other symptoms. There was still no sus-
picion of disease of the heart.
" On the 25th, however, there was
something in the stroke of the pulse which
it is impossible to describe, what I never
myself felt before, combined with a thrill,
almost a hiss, under the finger, more like
the vibration of a harp-string; it was
very remarkable in the right wrist, but
still more so in the left. The heart ivas
felt to be beating short, quick, and vio-
lently, 130 in a minute. On the appli-
cation of the finger to the space between
the second and third rib, a something be-
tween a crepitus of air in cellular mem-
brane, and the thrill at the wrist could be
discerned at every pulsation, that showed
us pretty clearly the real complaint. The
stethoscope was applied, and the preter-
natural rushing sounds very clearly heard.
About 16 ounces of blood were taken
from the arm ; it was cupped, but not
much, for the coagula were not firm, nor
was the buff very marked, being grey
rather than the usual yellow. I felt his
pulse that night while he slept, and it
was comparatively quiet and soft, having
lost all that hard jerking vibration it had
in the morning. 26th and 27th?Much
the same, pulse less violent, and No. 122.
On the 28th all the bad symptoms in-
creased ; he' was restless, anxious, and
evidently weaker; had no sleep, felt pain,
expressed a wish for nourishment, but
complained he could not eat; his pulse
was regular and less strong, but had the
same remarkable jerk and thrill. To-
wards the morning of the next day he
asked for the close-stool; he refused the
bed-pan, and got out of his bed and used
it. In half an hour after he had laid
himself down again, he died without any
struggle.
" On the 31st we examined the body.
On raising the sternum, the heart lay
more exposed to view than usual, less
enveloped in the lungs, of enormous size,
and occupying a more horizontal position
in the chest. There was no blood what-
ever in the cavities of the chest or peri-
cardium, but the serous fluid in the peri-
1.828] Hospital Pkactice. 475
cardium was in double quantity. On
looking attentively at the heart as it lay,
there appeared, between the origin of
the pulmonary artery and the arch of the
a?i'ta, a small tumour, of livid colour,
having a surface of pericardium, but evi-
dently going into ulceration. It felt solid,
but not hard. The right ventricle was
Very thin and flabby, the left as remark-
ably thick and firm and muscular. The
vessels were all in their natural state,
neither larger nor smaller nor disco-
loured. The interior of the right ventricle
Was natural. At the upper part of the
septum ventriculorum, just where the
edges of the semilunar valves mark the
origin of the aorta, an orifice appeared,
through which was escaping grey flakes
of coagulated lymph, which were followed
by clots of black blood, and some in a
fluid state. This tumour, when emptied,
Would contain a pigeon's egg; the orifice
Was ragged, and the interior surface'rough
and fibrous, like the cut surface of the
heart; it was not thin, nor pervious to
blood in any part. Two of the semilunar
valves were destroyed, one was entire.
Quaere. What was the cause of death in
this case ?"*
The foregoing case offers food for much
reflexion. We would ask Mr. Maynard,
in the first place, how it was that the
state of the vascular system, on the 29th
November, did not excite his attention to
the state of the central organ of the cir-
culation ? He examined the patient's ab-
domen, and found the pulse visible there
-?yet he did not examine the chest. Se-
condly, was the detraction of ten ounces
of blood from a young grenadier, with
the above-mentioned phenomena, a suf-
ficient depletion ? Thirdly, when he
says, on the 25th, that a something be-
tween a crepitus and thrill felt in one of
the intercostal spaces, " shelved pretty
clearly the real compluint," we would
ask him what was the real complaint
then, and how it was known by the
foregoing phenomena, as we are not
aware that the said phenomena afford any
data for a fixed diagnosis ? We do not
ask these questions from any captious or
hypercritical motives ; but to shew Mr.
Maynard and the public, that ausculta-
tion is not sufficiently attended to. The
complaint, in this case, was not suspected
till within three days of the patient's
death, and even then, we question whe-
ther a proper diagnosis was formed. The
extent over which the heart beat?the
impulsion?the difference of impulsion in
the two sides of the heart, were not in-
vestigated, (at least there is no mention
of them,) yet on them would chiefly have
rested the diagnosis, even on the first day
the patient entered the hospital. In res-
pect to the query at the end of the case?
we conceive that there can be very little
difficulty in concluding that immense hy-
pertrophy of the left side of the heart?
dilatation of the right chambers?valvu-
lar disease?and a tumour of the heart go-
ing into "ulceration," were quite enough
to destroy the life of even a grenadier of
the guards. In respect to the nature of
this tumour, we have no doubt that it
was a false aneurism, oi"aneurismal pouch
going off from between the heart and
aorta, such as we have described, in seve-
ral instances, in the 15th number of this
Journal. Considering the obloquy which
has been attempted to be thrown on aus-
cultation, of late, we have deemed it
necessary to comment on the foregoing
case, in order to shew the value of the
auscultic diagnosis, and the danger of
neglecting it.
2. ST. GEORGE'S HOSPITAL.
Bleeding in Erysipelas.*
The patient, a soldier, set, 38, was ad-
mitted Nov. 24th, 1827, in consequence
of a lacerated wound on the left side of
the forehead, and contusion behind the
ear, which he had met with four days
previously. There was a good deal of
action in the system, with head-ache and
costive bowels, for which he was bled to
? xxx. and took cal. grs. ij. p. jal. grs. vj.
4tis horis. On the next day, he was bled
again to ten ounces, and ordered salines
with sulphate of magnesia. On the 26th,
he had much pain in the head?general ex-
citement?pupils sluggish, and an erysipe-
latous blush appeared upon the forehead.
Lot. spt.fronti. V.S. ad ?xv. The ery-
sipelas extended with thirst, head-ache,
hard pulse, and he was again bled to
twenty ounces on the 27th. Next day
the erysipelas had extended over the right
* Med. Gazette, No. 6. p. 163. * Med. Gazette. No. 6. Jan. 12
470 Periscope ; or, Circumspective Review. [Jan. 26
cheek, with semi-delirium?quick and
full pulse?constant nausea. The blood
drawn on the preceding day was covered
with a thick layer of transparent fibrine.
Rep. V.S. ad ?xx. Cont. rned. Instead
of twenty, upwards of thirty ounces of
blood were taken by mistake, but during
the bleeding the erysipelatous blush faded,
and never afterwards returned, at least so
distinctly as before. The wounds on the
head healed, and, in the beginning of Jan.
the patient left the hospital cured.
Not very long ago, the surgeons of St.
George's Hospital were accused in the
Lancet, and that in pretty broad terms,
of killing their patients, by invariably
treating them, when labouring under ery-
sipelas, with bark! The case above nar-
rated forms, as our cotemporary justly
observes, an apt illustration of the vera-
city of that statement.
Popliteal Aneurism in both Legs?
Secondary Hemorrhage.*
This case, we understand, cxcited much
interest at the time, and furnished food
for no little misrepresentation and vitu-
peration in a certain quarter.
The patient was a carrier, tet. 53, ad-
mitted August 1st, with a large aneurism
in the left ham, which had commenced
twelve months previously. The tumour
had made considerable progress, and was
in one part exceedingly near the surface,
but not the least discolouration of the
integument was present. His health had
been always good, until the commence-
ment of the disease, at which time he
was much harrassed in body and mind
by a trial at law, since which he has been
very nervous and irritable. On his ad-
mission, he was labouring under a good
deal of fever, with great action in the
pulse and vascular system generally. On
inspecting the other ham, a distinct aneu-
rismal tumour was discovered pulsating
with extreme violence, and on the right
side of the neck the carotid was seen
beating very forcibly. Of the existence
of the second aneurism the patient was
not aware, and he was accordingly left
in ignorance of it. He was kept quiet
and bled until the 9th, when the opera-
tion was performed in the usual manner.
The ligature separated on the 23d, (four-
teen days after its application,) and at
this time the wound was little disposed
to heal, and the thigh was much swollen.
On the 6th September, (thirteen days
after the separation of the ligature,) se-
condary haemorrhage took place from the
wound?the bleeding could not be en-
tirely checked by the tourniquet, &c. and
on the morning of the Sth, Mr. Brodie
tied the artery at the groin. This liga-
ture remained until the 29th, (twenty-one
days,) and on the 11th October, (twelve
days subsequently,) secondary haemor-
rhage, to the extent of a pint and a half,
took place from the upper wound. The
patient was now in that state of irrita-
bility and excitement, that any further
operation was almost out of the question,
and, indeed, he positively declared that
he woidd undergo no more. Accordingly,
pressure, by means of properly adapted
trusses, pads, &c. was carefully employed,
but only with temporary effect, for on
the 30th bleeding took place, and, from
that time until the 2d November, when
he died, blood was continually oozing
from beneath the compresses, although
gentlemen were constantly sitting by the
poor fellow's side, day and night. The
immediate cause of death was mortifica-
tion, or rather a condition more nearly ap-
proaching to "dry gangrene" of the limb.
On dissection, it was found that the
ligature in the groin had been applied on
the superficial femoral, immediately be-
low the giving off of the profunda. The
artery at this point was entirely destroyed
by ulceration, so that no clot or adhe-
sions were discovered, and from this spot
to the site of the original operation both
artery and vein were converted into a
ligamentous undistinguishable mass. The
aneurismal tumour in the ham was solid,
no larger than an orange, and the vessels
passing to and from it obliterated. In
the other ham was a good sample of in-
cipient aneurism.
We could make some remarks upon
this case, did our limits permit. As it is,
we shall content ourselves with copying
from the Gazette, with a slight alteration
or two, the contradictions of the erro-
neous statements which have gone forth.
Lancet.?" Tumour remarkably tense and
solid."
The fact.-?It was soft, and could be almost
entirely emptied by pressure.
* Medical Gazette, No. 7-
1828] Hospital Practice. 477
Lancet.?" Afforded rather an indistinct
pulsation."
The fact.?The pulsation was visible two
or three beds off.
Lancet.?" Integuments about to ulcer-
ate."
The fact.?Not even a speck of disco-
louration had appeared.
Lancet.?" The man was kept at least a
fortnight in the hospital before
the artery was tied."
The fact.?He was admitted on the 1st,
and the operation was performed
011 the 9th. It was not done
sooner, because the patient had
much fever, and required repeat-
ed blood-letting and purging.
Lancet.?" The operation should have
been performed immediately on
the patient's admission."
The fact.?It should not.
Lancet.?" Mr. Brodie attempts to pass
a straight probe by main force
under, for we cannot call it
round the artery."
The fact.?Mr. Brodie attempts to do no
such thing. He uses the eye-
probe, simply because it is more
flexible than the common aneu-
rismal needle, and admits of any
curve required. The operation
was performed with great faci-
lity.
Now if this hospital report of the
Lancet's be not a tissue of the most
palpable blunders, we know not what is.
We have one or two cases by us at the
present moment, which will teach the
?Lancet what sort of stuff the " surgical
capability" of its reporters is made up of.
3. MIDDLESEX HOSPITAL.
Secondary Hemorrhage.*
J. Willmott, set. 37> prompter of Drury-
lane Theatre, had his left leg removed, by
amputation above the knee, for caries of
the tarsus, on the 10th October. The
case apparently did well, the ligatures
came away on the 20th, and the stump
had healed, save that two small sinuses
remained leading towards the bone. He
left the hospital on the 18th November,
and resumed his employment at the
theatre, one sinus still remaining open,
and the bone appearing likely to exfoliate.
On the 16'th December, the stump swel-
led and grew more painful, and on the
19th, a violent haemorrhage took place,
which was arrested by a ligature on the
limb. He was again brought to the
hospital, when Mr. Mayo tied the com-
mon femoral artery, distinctly above the
origin of the profunda. On tying the
ligature, the lower part of the artery con-
tinued to pulsate, though not so strongly
as before. Mr. Mayo thinking that the
knot might have slipped, applied a se-
cond ligature, but still the pulsation re-
mained. On removing the bandages
from the stump, there existed a sinus in
the direction of the femoral artery, and
a second opposite the bone, and through
them the blood must have issued. No
more haemorrhage ensued, but it was
necessary to remove some dead bone, and
on the 1st January, the ligature had not
separated from the vessel.
T. Bailly, ajt. 58, had his right leg
amputated above the knee, for ulcer and
caries of the bone, on the 13th November,
by Mr. Mayo. The vessels bled furiously,
and sixteen were tied, but in the evening
secondary haemorrhage came on, and it
was necessary to secure three more.
Next day there was great nervous irri-
tation, twitchings of the stump, &c.
from which he rallied for a time, but sank
on the 1st January. One ligature, in the
direction of the main artery, had not se-
parated, and from this spot hasmorrhage
had occurred which was restrained once
by pressure, and on its recurrence by the
actual cautery. Upon examination, the
bleeding was found to have proceeded
from a small artery upon the vastus in-
ternus, the femoral being firmly closed,
and containing a clot, an inch in length.
The plugging up of the vessel was found
to have depended on the adhesion of
the outer coat only, the inner coat not
having entered into the cicatrix in the
manner described by Dr. Jones. This ap-
pearance is very well shewn in a wood-cut.
Between these two cases, though simi-
lar in some respects, there is yet an ob-
vious difference. In the first, there was
no particular haemorrhagic tendency at
the time, and all went on favourably
enough, save that from weakness of
constitutional power, or other cause, a
sinus remained, and the ulceration sub-
* London Med. Gazette, No. 6, Jan. 12.
478 Periscope; or, Circumspective Review. [Jan.26
sequently extended to the artery. In the
second case, the hsemorrhagic disposition
was marked, and the patient seems to
have sunk more from the effects of what
took place on the day of operation, than
from the trifling bleeding which occurred
subsequently, though this, no doubt, was
the cause of great irritation to the system.
We have heard a comparison drawn be-
tween Mr. Mayo's first case, and that re-
lated above from St. George's Hospital,
but, to our minds, a most inappropriate
and unfair one. In the one case, there
was disease of the artery in a decidedly
aneurismal habit?in the other, there was
nothing of the kind.
4. ST. THOMAS'S HOSPITAL.
Fracture of the Seventh Cervical
Vertebra. *
Cases of fractured spine used to be con-
sidered as something uncommon in the
pages of a journal, but now, thanks to the
"gentlemen connected with the press,"
as Sir Richard Birnie has it, they are be-
coming plenty as blaek-berries.
H. C. set. 35, received an injury to the
spine, on the 1st of December, from a
sack of flour falling from .a height of 10
feet upon his shoulders. He was stunned
by the blow, and lost the use of his lower
extremities, but did not enter the hospital
until the 5th. The lower exti'emities, at
this time, were quite paralyzed, but he
could raise his arms to a certain extent
?breathing diaphragmatic?no priapism
?pain on attempting to move his head.
No displacement of vertebrae could be
discovered, so that Mr. Green was dis-
posed to consider the symptoms as depen-
dent on effusion. Cupping?injections,
and the catheter three times a day, were
employed, but the urine in a few days
became decidedly ammoniacal and offen-
sive, sloughs formed on the nates and
sacrum, and on the morning of the 23rd
the poor fellow died.
Dissection. The arch of the 7th cervical
vertebra was found to be broken, its right
inferior oblique process dislocated for-
wards to the extent of a quarter of an
inch, and its body fractured across. Up-
on the theca beneath the first dorsal arch
there was acoagulum of blood, whilst the
membrane here was vascular and dis-
tended. On exposing the medulla, much
serous fluid escaped, and the marrow it-
self opposite the last cervical vertebra
was of the consistence of creain.
This softening of the medulla Mr.
Green, in his clinical lecture, very pro-
perly considered as dependent on the in-
jury (and consequent inflammation) of ita
substance, rather than on any pressure
exercised by the bone. Mr. Green also
observed that, on the 8th day after the
patient's admission there were signs of
returning sensation in the lower extre-
mities, the patient being able to distin-
guish which foot was touched ; but on
the following day these good symptoms
had disappeared. This, Mr. Green was
disposed to attribute to the setting up of
inflammatory action, which induced a
temporary excitement in the spinal mar-
row, and temporary return of sensibility
in consequence. The reporter, whilst he
appears to be struck by this " ingenious
explanation" of Mr. Green's, (an expla-
nation, by the bye, which was given long
ago by Mr. Charles Bell, in his celebrat-
ed controversy with Sir Astley Cooper,)
declares pretty plainly, that Mr. G. la-
boured under an "illusion" as to the
fact of the amendment having taken place
on the day mentioned. The reporter ex-
amined the man soon after Mr. G. and
found, "to his complete satisfaction, that
there teas no return of sensibility." As
the shock to Mr. Green's nervous system
must be very severe indeed upon reading
this, the reporter has very judiciously and
humanely endeavoured to allay the con-
stitutional irritation, by declaring that
what he did, was only " with a view of
establishing truth," and preventing the
dissemination of so dangerous a doctrine,
as that H. C. could tell which toe was
pinched upon the eighth day 1
5. ST. BARTHOLOMEW'S.'
Malignant Bleeding Polypus of the
1 Nose.
An interesting case of this terrible disease
is detailed in No. 6 of the Gazette. We
must refer our readers for the details,
which are exceedingly well given, to the
journal in question; but we may just
* Lancet, No. 228, Jan. 12.
1828] Hospital Practice. 479
state, that it had existed for eighteen
months, had been removed by the forceps
twenty-three different times, and was con-
stantly bleeding. After putting the pa-
tient under proper diet, &c. Mr. Earle
attempted to dissect it out by an incision,
commencing at the top of the right nasal
bone, and extending through the ala nasi.
The polypus was attached to the nasal
and maxillary bones, which were rough,
and their Schneiderian membrane thick-
ened. Each stroke of the knife was fol-
lowed by profuse haemorrhage, and it
was thought necessary to" remove part of
the nasal and maxillary bones, and to ap-
ply the actual cautery. The operation,
which does no discredit to Mr. Earle,
was performed on the 29th September,
and, by the 30th October, the fungus had
again made its appearance. It went on
increasing in size, bled profusely, and,
on the 30th Dec. the patient died.
On dissection, the fungus appeared to
have taken its origin from the membrane
covering the vomer, superior turbinated
bone, and cribriform lancella of the asth-
moid. The lungs, the liver, the spleen
and pancreas, the renal capsules, fat sur-
rounding the bladder and rectum, &c. &c.
were studded with tumours, some of them
medullary, some of them resembling clots
of blood, and readily breaking down un-
der the fingers.
Strangulated Exomphalos in the
Eighth Month of Pregnancy.*
E. Wright, ret. 31, had been always sub-
ject to a tumour at the navel, which,
about the 4th or 5th month of pregnancy,
invariably swelled and became painful,
but subsided under the use of a purgative.
On the 30th Sept. being then 7 or 8
months gone with child, she was seized
with pain in the umbilicus and vomiting,
which, not being relieved by castor oil,
she entered the hospital, on the 1 st Oct.
under the care of Mr. Lawrence. The
hernial tumour was soft, irregular, not
very tender, and consisted principally of
omentum, whilst there was no tenderness
whatever of the abdomen. Castor oil,
leeches, taxis, &c. were employed in vain,
and Mr. Lawrence, notwithstanding the
mildness of the symptoms, determined
on operating. On laying open the sac, a
quantity of omentum, and a small portion
of discoloured intestine, were exposed.
The latter was returned, and a part of
the omentum removed, one or two small
vessels requiring a ligature. She had
four or five motions after the operation,
but, next morning, there came on inflam-
mation about the wound, for which she
was bled to sixteen ounces?had twenty
leeches to the abdomen, &c. On the 3d,
the bleeding and leeches were repeated,
and a poultice applied to the wound.
Oct. 4th. Better, V.S. ad ?xiv. An
abscess formed " on the tumour," (this
means, we suppose, in the omentum)
which was opened, and the patient did
very well.
It is not many years since recovery
from the operation for hernia, at some of
our hospitals, was no very common oc-
currence, whilst at present, when circum-
stances are tolerably favourable, patients
as rarely sink under or after it. This
change in the comparative results of the
operation, we have no hesitation in as-
cribing to the circumstance of surgeons
resorting to it much earlier than they
used to do. In the case, however, under
review, we are not quite sure that the
protrusion might not have been returned
without the operation, for the symptoms
were exceedingly mild, and the reporter
makes no mention whatever of the state
of the stricture. It may have been firm,
it may have been lax, or there may have
been no stricture at all, from any thing
which appears to the contrary on the face
of the report, although the direction of
the cuts, the sutures, and the straps, are
detailed with the most laudable fidelity.
We observe that in this, as in most other
instances, Mr. Lawrence removed a por-
tion of the omentum, and certainly, as
far as the case goes, without any ill effect.
We should be glad to hear the results of
Mr. Lawrence's experience on this point,
as it is one of much interest, and a good
deal of difficulty.
College of Physicians v. Dr. Harrison.
We were right in our information, that a
committee had been appointed to collect
proofs of Dr. Harrison's practice. They
have effected their purpose (as they be-
* Lancet, No. 228,
480 Periscope ; ou, Circumspective Review. [Jan. 28
lieve) and notice of action hasbeen served.
We said that, had we been in Dr. Harri-
son's place, we should have given up the
admission of practice at once, and thus
met the action boldly. Dr. H. by halt-
ing in the commencement of the battle,
has lost the " vantage ground," as far as
concerns himself personally, without gain-
ing any adequate security of position by
this ill-timed caution. This, however,
makes no difference in the general result
of the contest. Nothing will give us
greater pleasure than the fact of a verdict
being gained by the College. It is just
what we ardently hope for. While an
unjust law slumbers, it is safe. The
moment it is put in execution, the process
of abrogation commences. A statute
originally designed for the suppression of
quacks, is now turned against regular
physicians of the first medical university
in the united kingdom. We had little
idea that the council of the College would,
in the year of our Lord, 1828, prove the
truth of the ancient adage?
" Quem Deus vult perdere, prius dcmentit."
Anxious for the establishment of libe-
ral institutions and justice, we shall
restrain ourselves onthe present occasion,
lest we should disturb the natural course
of events. Mr. Brougham will remove
the veil, and the picture which will be
drawn of the state of this branch of the
profession, in a court of law, will open
the eyes of statesmen?while the general
indignation of the Profession, at such a
flagrant insult to the diplomas of the
Edinburgh, and other regular universities,
will lead to the desired object?a general
investigation of the state of the profes-
Libehahty and Illiberaltty.
We were much grieved to see a letter,
lately published in the Medical Gazette,
from a correspondent of Dr. A. T. Thomp-
son, reflecting, in rather illiberal terms,
on foreign schools of medicine. Those
who know how openly and hospitably
English students and practitioners are
received in the public schools and insti-
tutions of the Continent, cannot but de-
plore the impolicy and narrow-mindedness
of individuals, who, from hasty and crude
views of things, permit themselves to in-
jure their countrymen, and the whole of
the English medical profession, in the
eyes of liberal foreigners, by such preju-
diced and unfounded statements as those
alluded to.
But what shall we say to the disgrace-
ful scenes that are now exhibiting in the
medical press, in respect to the injuries
sustained by the medical officers of public
institutions from the misrepresentations
of false reporters? Let any man read
the deluges of abuse which have been
poured on Mr. Earle and Mr. Keate, in
the last Number of the Lancet, for hav-
ing merely corrected the wilful falsehoods
of ignorant reporters, and say whether
or not the Saturnia regna are returning
in this country! The Lancet is playing
a desperate game. It seems determined
to try how far the most vulgar ribaldry
and Billingsgate slang can be crammed
down the throats of a learned and liberal
profession, as a substitute for knowledge,
candour, and practical information. Thus,
whole columns of that journal are filled
with the most low-lifed and disgusting
epithets, lavished on men of acknow-
ledged probity, talents, and honour, while
all their statements are distorted, garbled,
and misrepresented, so as to make a few
of the very lowest and most ignorant of
the profession laugh, and all the sensible
and ingenuous grieve?if they can permit
themselves to wade through such detes-
table trash ! We confess, however, that
we are not sorry to see justice, good sense,
and proper feeling, thus violently out-
raged. It is precisely in this way that
evil works its own ruin. Whatever may
be the proportion in which good and bad
principles are mixed in human nature, a
time always arrives, vvhen the former is
approved, and the latter spurned. That
period is rapidly approaching, if we be
not much mistaken. At all events, the
advocates of the evil principle are taking
the most direct paths to bring about the
triumph of the good. We would advise
the advocates of the latter to oppose a
calm, but steady resistance, in the detec-
tion of error and misrepresentation, trust-
ing to the good sense of Englishmen, and
the justice of their cause, for a final and
signal victory.
"When Peace and Mercy, banished from the plain,
Sprang on the viewless winds to Heaven again-
All ?all, forsook the friendless, guilty mind-?
But HOPE, the channcr, lingered still behind t"

				

